# Identifying Premature Ventricular Complexes from Outflow Tracts Based on PVC Configuration: A Machine Learning Approach

**DOI:** 10.3390/jcm12175558

**Published:** 2023-08-26

**Authors:** Sargun Bajaj, Matthew T. Bennett, Simon W. Rabkin

**Affiliations:** 1 Faculty of Medicine, Vancouver Hospital Cardiology, Vancouver, BC V5Z 1M9, Canada; sargun23@ubc.ca (S.B.);; 2 Department of Medicine (Cardiology), University of British Columbia, Vancouver, BC V5Z 1M9, Canada

**Keywords:** premature ventricular complexes, PVC morphology, outflow tract origin, cluster analysis, unsupervised machine learning

## Abstract

Background: Current inferences about the site of origin (SOO) of premature ventricular complexes (PVC) from the surface ECG have not been subjected to newer data analytic techniques that identify signals that are not recognized by visual inspection. Aims: The objective of this study was to apply data analytics to PVC characteristics. Methods: PVCs from 12-lead ECGs of a consecutive series of 338 individuals were examined by unsupervised machine learning cluster analysis, and indexes were compared to a composite criterion for SOO. Results: Data analytics found that V1S plus V2S ≤ 9.25 of the PVC had a LVOT origin (sensitivity 95.4%; specificity 97.5%). V1R + V2R + V3R > 15.0 (a RBBB configuration) likely had a LVOT origin. PVCs with V1S plus V2S > 12.75 (LBBB configuration) likely had a RVOT origin. PVC with V1S plus V2S > 14.25 (LBBB configuration) and all inferior leads positive likely had a RVOT origin. Conclusion: Newer data analytic techniques provide a non-invasive approach to identifying PVC SOO, which should be useful for the clinician evaluating a 12-lead ECG.

## 1. Background

The assessment of premature ventricular complexes (PVCs) is the focus of renewed interest because of the potential of PVCs to induce cardiomyopathy [1] and be a predictor of potentially fatal cardiac arrhythmias and sudden death [2,3,4]. In a meta-analysis of 11 studies comprising 106,195 individuals from general populations, frequent PVCs were associated with a substantial increase in the risk of cardiac mortality, especially sudden cardiac death [4]. The technology to ablate the cardiac focus of PVCs has the potential, in some circumstances, to mitigate the adverse long-term outcome associated with PVCs [1]. The presence of a high PVC burden is a recommendation for PVC ablation if PVCs cannot be managed by antiarrhythmic drug therapy, especially if there is a single dominant PVC morphology [5], which suggests a single site of origin. Thus, defining the site of origin of PVCs is important in order to assess not only the risk of the PVC but also the risk and potential benefits of a PVC ablation strategy.

PVC characteristics have been proposed for a long time to aid in the identification of the site of origin of PVC and/or ventricular tachycardia [6,7,8,9]. PVC configuration has been used to predict an origin in the left ventricular outflow tract (LVOT) or right ventricular outflow tract (RVOT), both of which represent the most common types of idiopathic ventricular arrhythmias [10,11,12,13]. A number of different criteria have been proposed to identify the site of origin of PVCs. Amongst these, three algorithms are of interest because they have undergone assessment of sensitivity and specificity for the determination of RVOT or LVOT origin of PVCs or ventricular tachycardia [14,15,16]. However, each of these three algorithms advanced different criteria [14,15,16]. Furthermore, all of them were based on a single clinical criterion from an ECG at the time of PVC ablation [14,15,16].

Newer technological approaches utilizing unsupervised machine learning, such as cluster analysis, seek inherent patterns in data sets that are useful in many disciplines, including cardiology [17]. This kind of methodology is not only a less ‘biased’ approach but can also identify signals that are not recognized by clinical visual inspection of the ECG [18]. The primary objective of this study was to utilize unsupervised machine learning to develop newer criteria to define PVC characteristics that can assist in identifying the site of origin of PVCs [14,15,16].

## 2. Methods

### 2.1. Case Selection

All ECGs at our institution that were interpreted as having a premature ventricular complex (PVC) by an interpreting cardiologist during a 9-week period were examined, and one ECG per patient was selected for evaluation. Thus, the study consisted of a consecutive series of patients with PVCs on a 12-lead ECG. The study was approved by our institution’s (the University of British Columbia, approval code: H19-03720) Research Ethics Board. 

The exclusion criteria included the presence of acute myocardial infarction, atrial fibrillation, atrial flutter, pacemaker rhythm, fusion beats, complete left or right bundle branch block, complete AV block, or ventricular tachycardia. ECGs were also excluded if there were significant baseline artifacts that prevented accurate identification of an isoelectric line.

### 2.2. Electrocardiogram Measurements

A total of 12-lead electrocardiograms were recorded digitally on Muse™ version 9.0 SP6 (General Electric, Boston, MA, USA). The ECGs were viewed on a 10-second ECG at 25 mm/s paper speed. Data extracted from the 12-lead ECG of the PVC included 3 categorical variables: frontal plan axis (FPA), transition lead (TL) in precordial leads, and bundle branch block pattern in precordial leads. For FPA, PVC axes was categorized into normal axis (NA), left axis deviation (LAD), right axis deviation (RAD), and extreme axis deviation (EAD). The normal axis was defined as FPA between −30° and + 90°. LAD was defined as FPA between −30° and −90°. RAD was defined as FPA between + 90° and + 180°. EAD was defined as FPA between −90° and −180°. Borderline FPA were −90°, + 90°, −30°, + 180°or that could not be assigned to one of these 4 categories of frontal plane axis.

TL was defined as the precordial lead that was isoelectric or the precordial lead where amplitudes changed from positive to negative. TLs were categorized as V1, V2, V3, V4, V5, and V6, corresponding to the precordial leads where this transition occurred. If there was no isoelectric lead, the transition was defined as occurring between two leads. Thus, TLs were categorized as V1.5, V2.5, V3.5, V4.5, and V5.5 when the transition occurred between lead V1 and V2, Lead V2 and Lead V3, Lead V4 and Lead V5, and Lead V5 and Lead V6, respectively. One person was trained on the measurement methods and made all measurements in order to eliminate interobserver variability. There was minimal variability as the measurements were made by the computer ruler function on Muse™ version 9.0 SP6 (General Electric, Boston, MA, USA).

The presence of a BBB pattern of the PVC was determined by evaluating the R wave progression in the precordial leads. When PVC R wave progression was initially positive in the right precordial leads (V1 to V3) and negative in the lateral precordial leads (V4 to V6), the PVC was considered to have a Right bundle branch block (RBBB) pattern. If the reverse was found, the PVC was considered to have a left bundle branch block (LBBB) pattern. If all R waves in the precordial leads were predominantly positive or predominantly negative, the PVC was considered to have a posterior or anterior origin, respectively. 

PVC amplitudes were measured as the absolute magnitude of the R wave in leads V1 (V1R), V2 (V2R), and V3 (V3R), and S in leads V1 (V1S) and V2 (V2S). The absolute magnitude of the R or S wave was defined by first determining the isoelectric line from the T to P segment (T wave to P wave) and measuring the mV from the isoelectric line to the peak of the S or R wave.

### 2.3. Determination of the Site of Origin of PVC

The site of origin of the PVCs was based on a composite of the following three formulas that have been evaluated for their capacity to predict RVOT or LVOT origin specifically:

(i) (a) RVOT origin (V1S – V2S) − (V1R + V2R) > 1.625
  (b) LVOT origin (V1S − V2S) – (V1R + V2R) ≤ 1.625 [14].
(ii) (a) RVOT origin: V2 transition ≤ 0.6 (LBBB with transition in lead V3)
  (b) LVOT origin: V2 transition ratio ≥ 0.6 [15]
(iii) (a) RVOT origin: V2S/V3R > 1.5
  (b) LVOT origin: V2S/V3R index ≤ 1.5 [16];

Each of these formulas were applied to the data for each patient’s ECG. An arithmetic mean (average) was calculated from the three criteria for presumed RVOT and LVOT origins.

### 2.4. Data Analysis

Data are presented both as the mean ± SD or median (Quartile 1–Quartile 3). Cluster Analysis was performed using R Studio Version 1.3.959. Cluster analysis used the partition around mediods methods (PAM). The Cluster analysis was first performed on all the data and then on the basis of whether the PVC was classified as a right or left bundle branch block configuration. Next, within each bundle branch pattern, the clustering was also performed in two subsets of samples: (i) all inferior leads were positive and (ii) not all inferior leads were positive (see Supplement). Silhouette width is a measurement based on the closeness of an object to its own cluster, with a higher magnitude indicating a higher relative quality of clusters [19]. The silhouette width for each cluster analysis is used to identify the optimal number of clusters [20]. In order to cross validate the Cluster analysis that used the PAM method, we used Weka, which partitions the observations into k clusters, with each observation being in a cluster based on its proximity to the cluster center (Simple K-means) [21].

After the cluster analysis, an equation was selected to optimize sensitivity and specificity and determine whether the PVC belongs to one cluster or another. Sensitivity was calculated by dividing the total number of PVCs in the cluster specified that met the criteria by the total number of PVCs in that cluster. Specificity was calculated by dividing the total number of PVCs in the cluster that did not meet the criteria by the total number of PVCs in that cluster.

### 2.5. Statistical Analysis

Tests of significance use parametric testing. To determine significant differences between clusters formed using Partitioning around medoids, independent *t*-tests were performed when there were only two clusters and ANOVA when three clusters were compared.

## 3. Results

The sample size consisted of 338 patient ECGs that met the inclusion criteria after screening a total of 436 ECGs, with the difference being those meeting the exclusion criteria. PVC characteristics evaluation showed that 48.2% had a PVC with a LBBB pattern, 27.5% had a PVC with a RBBB pattern, 18.9% had a posterior pattern, and 5.3% had an anterior pattern (Table S1). The normal axis was the most common frontal plane axis and was present in 37.6% of cases. The QRS transition lead was most frequently between V3 and V4.

Cluster analysis utilizing PAM on the entire sample, including categorical variables: BBB, transition lead, and frontal plane axis, did not produce significant differences between each of the three clusters (Table S2). Subsequently, cluster analysis of the entire PVC sample was performed after removing the categorical variables (Table 1). It produced two clusters with a silhouette width of 0.327. A 2D graph of the clusters demonstrated the separation (Figure S1). Six out of eight numerical variables were significantly different between the two clusters (*p* < 5 × 10^−13^). Eighty-two percent of PVCs in Cluster 1 had an RVOT site of origin, based on the three equations, whereas 92.6% of PVCs in Cluster 2 had an LVOT site of origin. In order to cross validate the Cluster analysis that used the partition around mediods method, we used another machine learning methodology, namely Weka, that partitions the observations into k clusters, with each observation being in a cluster based on its proximity to the cluster center (K-means) [21]. The number of PVCs in each cluster using both PAM clustering and Simple K-means was not significantly different (*p* = 0.23). To derive a simplified approach to characterizing Cluster 2 or the LVOT origin of a PVC, several equations were examined. We found that if the sum of V1S and V2S is ≤ 9.25, the PVC will be Cluster 2 with a sensitivity of 95.4% and specificity of 97.5%.

Cluster analysis of a PVC sample with a RBBB pattern without any categorical variables produced two clusters with a silhouette width of 0.261. (Table 2). The differentiation can be visualized using a 2D representation (Figure S2). Seven of eight numerical variables were significantly different between the two clusters (*p* < 0.05). Overall, Cluster 1 and Cluster 2 had 100% and 93% PVCs displaying LVOT origin, respectively. For PVCs with a RBBB configuration, the formula V1R + V2R + V3R > 15.0 identified cluster 1 with a 95.9% sensitivity and a 90.7% specificity. There were no significant differences in the average values of the 8 numerical variables in both clusters using the partition around mediods method compared to the Simple K-means method.

Cluster analysis of a PVC sample with a LBBB pattern without any categorical variables produced two clusters with a silhouette width of 0.314 (Table 3). The differentiation was visualized using a 2D representation (Figure S3). Six of eight numerical variables were significantly different between the two clusters (*p*< 0.01). Cluster 1 had a majority LVOT (63.6%), and Cluster 2 had mostly RVOT origin (82.7%). V1S + V2S > 12.75 predicted assignment to Cluster 2 with a sensitivity of 94.9% and a specificity of 95.2%. There were no significant differences in the average values of the eight numerical variables in clusters using the partition around mediods method compared to the Simple K-means method. Cluster analyses of PVC samples with LBBB pattern and superior or inferior axes, using partitioning around mediods, identified two clusters each that separated LVOT origin from RVOT origin (Table 4 and Table 5). There were no significant differences in the average values of the eight numerical variables in clusters using the partition around mediods method compared to the Simple K-means method (data not shown).Other analysis is presented in tabular form the supplement (Tables S3–S5).

## 4. Discussion

This study has made several novel contributions to characterizing premature ventricular complexes. The 12-lead ECG has been said to have the potential to be ‘invaluable’ in determining the origin of PVCs and, in so doing, identifying the site of origin of potentially fatal ventricular arrhythmias [22]. Traditionally, clinicians have relied on assessing whether the PVC had a bundle branch configuration and evaluating its frontal plane QRS vectors to suggest the PVC’s site of origin [8,9,10,13,23]. Then measurements of QRS deflections and/or the precordial lead of QRS transition were used [14,15,16] to suggest the site of origin. While there are no survey methods for which approach predominates, it appears that current clinical assessment of the site of origin of PVCs relies on consideration of the 12-lead ECG characteristics, including the bundle branch block pattern, axis, QRS polarity in lead V6, maximal deflection, QRS duration, and precordial transition [24]. In contrast to the current clinical pattern recognition approach, we used a data agnostic approach, namely machine learning technology in the form of cluster analysis with Partitioning around medioids, to categorize 338 persons’ PVCs into various distinct groups.

Machine learning is usually divided into two kinds: supervised learning, where the algorithm is trained on datasets to map known inputs and outputs, and unsupervised learning, where the algorithm processes unlabeled datasets to find patterns for classification [25]. We used unsupervised machine learning and applied cluster analysis, which has recently been shown to be effective in cardiology, especially in heart failure, where it has permitted the identification of subgroups with different outcomes [26,27].

Machine learning approaches have been used to detect the presence of PVCs [28]. One kind of machine learning: deep learning, utilizes deep neural networks and can be either supervised or unsupervised [25]. Application of that approach to the 12-lead ECG has outperformed standard clinical ECG criteria for the diagnosis of left ventricular hypertrophy and can predict the subsequent occurrence of cardiac sudden death in patients after an acute cardiac event [29,30]. The application of machine learning to the ECG has allowed for the identification of patients in sinus rhythm who subsequently develop atrial fibrillation [31]. It has also been identified from the ECG, patients who have ventricular dysfunction (ejection fraction ≤ 35%) [18].

With this background, we sought to learn whether analysis of an aspect of a PVC can provide information about PVC morphologies. The unsupervised machine learning approaches identified several different PVC configurations or morphologies. Cluster analysis used the partition around mediods methods (PAM). This cluster approach was further validated by employing a different cluster analysis methodology, Simple K-means using Weka. The two approaches found similar clusters.

**Figure 1:**
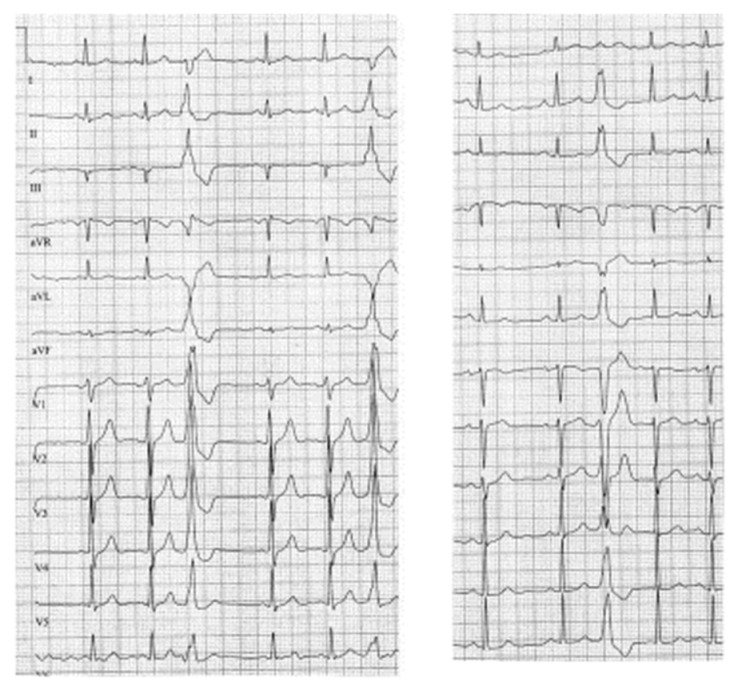
Shows different ECGs with PVCs with a Right Bundle Branch Block configuration (**left panel**) and a Left Bundle Branch Block configuration (**right panel**), from which detailed QRS measurements can be made.

**Figure 2:**
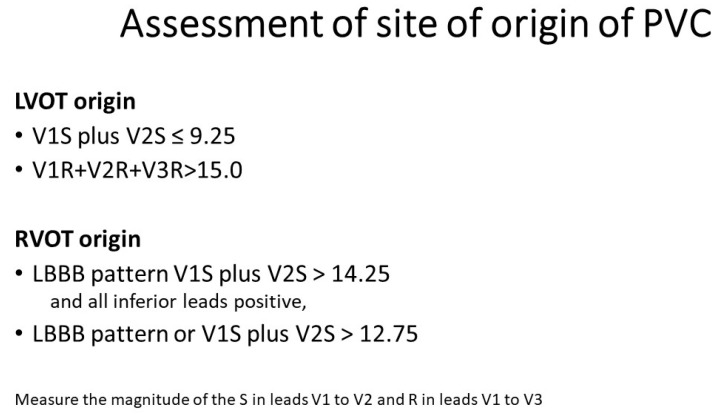
Algorithms for the identification of the site of origin of PVC.

We next assessed whether the identified clusters predicted their putative site of origin. We were able to predict SOO for PVCs regardless of whether they had an LBBB or RBBB pattern (Figure 1). Our first cluster analysis with the entire sample excluding categorical variables (LBBB or RBBB pattern) showed that the two clusters predicted RVOT vs. LVOT, with Cluster 1 mainly having a presumed RVOT origin and Cluster 2 mainly consisting of PVCs with a presumed LVOT origin. This cluster analysis approach was further validated by the use of a different cluster analysis methodology, Simple K-means using Weka, which found similar clusters. The equation V1S + V2S ≤ 9.25 estimates an LVOT site of origin with a sensitivity and specificity of >90%. The next best approach was the sum of R waves in V1 plus V2 plus V3 greater than 15.0. A RVOT origin of a PVC can be inferred when the sum of the S waves in V1 plus V2 is greater than 14.25 with all inferior leads being positive or when the sum of the S waves in leads V1 and V2 is greater than 12.75. Our equations simplify the approach as they can be independent of decision making about which type of BBB pattern exists. Importantly, it is based on the data agnostic approach of machine learning (Figure 2).

PVC characteristics have been previously proposed to aid in the identification of the site of origin of PVC and/or ventricular tachycardia [6,7,8,9]. PVC configuration has been used to create algorithms that help predict specific origins in the LVOT or RVOT, which represent the most common types of idiopathic ventricular arrhythmias [10,11,12,13]. Previous studies, however, have not always used current data analysis techniques to characterize PVCs. Identifying the correct site of origin for PVCs has many clinically significant advantages [9]. Determining the PVC location can alert the clinician to PVCs that may be associated with underlying cardiomyopathy and prompt them to intensify investigations and follow-up. Furthermore, when clinicians pursue investigations for cardiomyopathy through imaging (echocardiogram or MRI), they can instruct the imager to scrutinize specific areas of the myocardium for scars. Lastly, accurate PVC location ascertainment will allow clinicians to counsel patients regarding the risk and success of ablation depending on whether PVCs are in the left or right ventricle, epicardial or endocardial, and their proximity to the coronary arteries or the aortic valve. PVC localization to the RV outflow tract is easier for ablation, while some LVOT outflow tract ablations have a greater risk of damage to the aortic valve or left main coronary artery.

### Study Limitations

There are several limitations to our study that warrant consideration. First, we infer the site of origin of the PVC and do not have electrophysiological data to test the validity of our clusters or confirm the site of PVC origin. While there have been a number of previous studies that developed approaches to estimate the site of origin of PVCs [7,9,13,32,33], we used three equations, well recognized as proxies for electrophysiology data on the origin of outflow tract PVCs [14,15,16]. Second, attempts to validate each of our formulas are difficult. Therefore, we validated our principal clusters by performing another type of cluster analysis. For the entire sample, clusters formed using PAM were similar to clusters formed using Simple K-means, as the number of PVCs in each cluster was not significantly different between the two methods. Additionally, the average values for each of the eight numerical variables in both clusters were not significantly different between the two methods. These results were replicated for PVC samples with the LBBB pattern only but were not replicated for PVCs with the RBBB pattern. This suggests that clustering formed with PAM clustering and Simple K-means was similar for both the entire PVC sample and for LBBB pattern PVCs but not for RBBB pattern PVCs. Third, some investigators contend that ECG criteria for differentiating right from left ventricular outflow tract ventricular arrhythmias and for localizing ventricular arrhythmias within the aortic sinus of Valsalva have limited accuracy [34]. Recognizing this challenge, we used not one but three different equations that were derived from studies that each used invasive cardiac electrophysiological techniques to identify and ablate the arrhythmia [14,15,16]. Each of these approaches is in clinical use to suggest a site of origin for ventricular ectopics. Nevertheless, one must be mindful that all site of origin algorithms faces the challenge that the outflow tract is a ‘complex region of tissue overlaps where factors such as cardiac rotation, preferential conduction patterns, variation in myocardial fiber orientation, shared myocardial connections bridging left- and right-sided outflow tracts, and wave-front summation make differentiation of the SOO less reliable’ [35]. Nevertheless, SOO criteria are frequently used in clinical ECG interpretation and clinical patient assessment. Importantly, there are few other markers that can provide an initial guide to the PVC location for an ablation procedure. Thus, assessment of PVC morphology or configuration is a useful guide that must also take into consideration the complexities inherent in attempting to localize SOO. Further research, however, should be conducted to examine these algorithms in cardiac electrophysiological studies. Fourth, it is difficult to state with precision the generalizability of our findings when applied to a larger population. This study attempted to minimize bias by having a relatively large data set (larger than any of the other three studies that formed the algorithm on which the research was compared). Importantly, the patient sample represents a consecutive series of cases that fulfilled the entry criteria but did not meet the exclusion criteria. Consequentially, it should be readily extrapolated to the population of a large tertiary/quaternary care hospital. How it would apply to individuals with PVCs in a community setting needs to be defined. Fifth, the criteria for site of origin were already validated in each of the three studies using the surface ECG at the time of localization of the site of origin by catheter ablation. To further minimize bias, the site of origin was an average of the three different algorithms. It is not possible to do an AUC data analysis because there is no outcome factor to analyze against. The site of origin was the mean value of three equations for each of the Right and Left outflow tracts. We do not know the accuracy of the composite average. A novel contribution of this manuscript is that the unsupervised machine learning approach identified mainly two types of PVC complexes. It is generally recognized that PVCs usually originate from the right or left ventricular outflow tract, so our findings are consistent with known knowledge but extend it because it does not require labelling PVCs as right or left BBB. The experimental design did not have a training and test group because the primary objective was to determine if the machine learning approach could distinguish different characteristics of PVCs. The next objective was to determine whether the different characteristics correlated with the site of origin. As the site of origin was a composite of three studies, there was no direct assessment of the site of origin to make a precise assessment. The next research efforts should have a direct stimulation of different sites in both ventricles and correlate it with the data obtained in this study. A study of stimulation or pacing multiple sites in both ventricles is a challenging undertaking that requires further planning and work. 

## 5. Conclusions

**Figure 3:**
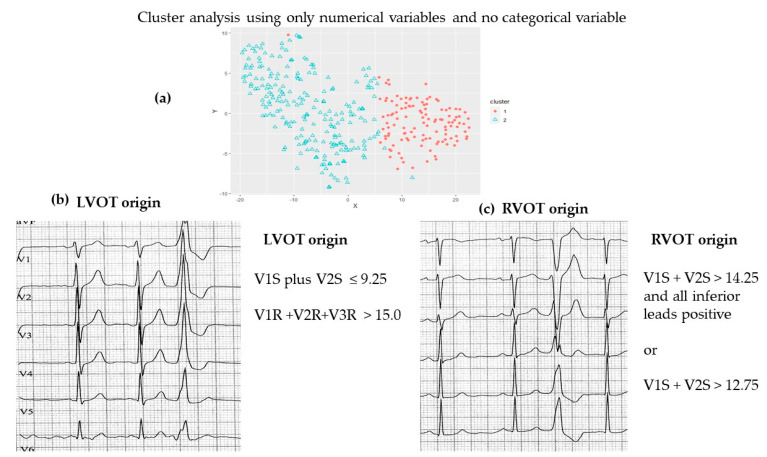
Graphical abstract shows: The separation of ECG PVC characteristics into two types as a result of cluster analysis using only numerical variables and no categorical variables (**a**) PVCs with a Right Bundle Branch Block configuration with criteria for LVOT origin (**b**) and PVCs with a Left Bundle Branch Block configuration (**c**) with criteria for LVOT origin .

Consideration should be given to abandoning the practice of simply describing the presence of a PVC on a 12-lead ECG and replacing it with a detailed analysis of the QRS complex. Using the new approach of unsupervised cluster analysis on a large sample, we found that the sum of V1S + V2S less than or equal to 9.25 of a PVC suggests an LVOT origin of the PVC, while large values of the sum of the S wave amplitude in V1 and V2 suggest a RVOT origin (Figure 3). Published algorithms have been critiqued because of their intricate computational methods [13]. Our approach eliminates the need to decide whether the PVC has a RBBB or LBBB configuration or requires the exclusion of those with an anterior or posterior ‘pattern’. Not only does this study provide new formulae with a simple method of calculation and high levels of accuracy in identifying the site of origin for PVCs, but it is also based on the data-agnostic or unbiased approach of unsupervised machine learning. Our approach should aid clinicians in scrutinizing specific areas of the myocardium for scars and assist in counseling patients about the risk and success of ablation that are dependent on the site of origin of the PVC.

### Future Prospects

The algorithms identified in this study should be incorporated into computerized ECG interpretation programs. Data agnostic or unbiased approaches such as unsupervised machine learning hold considerable potential to improve cardiology care because they seek inherent patterns in large data sets that challenge humans ability to do so. The same machine learning approach should be applied to infrequent sites of origin of PVC, such as papillary muscles. This approach to defining the site of origin of PVCs should next be tested for its ability to identify individuals at different risks for cardiac morbidity and mortality, especially sudden death.

## Figures and Tables

**Table 1:** Cluster analysis on the entire sample based on only numerical variables using partitioning around mediods (left) and Simple K-means clustering (right).

	Partitioning around Mediods			Simple K-Means	
	Cluster 1	Cluster 2	*p*	Cluster 1	Cluster 2
V1S	6.50 (5.13–8.00)	1.32 (0.5–2.00)	<2.2 × 10^−16^	6.71	0.98
V2S	9.00 (7.00–12.38)	1.25 (0.75–2.50)	<2.2 × 10^−16^	9.33	1.73
V1R	0.50 (0.00–1.25)	3.00 (1.5–5.25)	<2.2 × 10^−16^	0.73	3.78
V2R	1.25 (0.50–2.45)	5.75 (3.50–8.50)	<2.2 × 10^−16^	1.70	6.48
V2R Sinus	1.75 (1.00–3.00)	2.00 (1.00–2.26)	0.6941	2.14	2.29
V2 Total PVC	11.00 (8.50–14.25)	7.75 (5.00–9.80)	5.587 × 10^−13^	11.03	8.16
V2 Total Sinus	8.50 (6.00–10.38)	7.75 (5.63–10.00)	0.1767	8.54	8.23
V3R	2.00 (1.00–3.50)	6.50 (4.00–9.50)	<2.2 × 10^−16^	2.77	7.15
Transition Lead	No transition lead: 1 V2.0: 2 V2.5: 21 V3.0: 5 V.35: 36 V4.0: 11 V4.5: 14 V5.0: 5 V5.5: 6 V6.0: 2	V1.0: 5 V1.5: 31 V2.0:12 V2.5: 24 V3.0: 3 V3.5: 23 V4.0: 4 V4.5: 13 V5.0: 16 V5.5: 14 V6.0: 8			
Frontal Plane Axis	NA: 70 (58.8) RAD: 17 (14.3) LAD: 26 (21.8) +90: 5(4.2) −30: 0(0) −90: 0(0) +180: 1(0.8)	NA: 57 (26.0) RAD: 53 (24.2) LAD: 67 (30.6) EAD: 24 (11.0) +90: 10 (4.6) −30: 3 (1.4) −90: 5 (2.3) +180: 0 (0)			
BBB	LBBB: 102 (85.7) RBBB: 0 (0) Posterior: 2 (1.7) Anterior: 15 (12.6)	LBBB: 61 (27.9) RBBB: 93 (42.5) Posterior: 62 (28.3) Anterior: 3 (1.4)			
Average percentage of RVOT based on 3 equations	82.3	7.4	<2.2 × 10^−16^		

Values were reported as median (Q1–Q3) or count (%). The number of clusters is based on the relative silhouette width. Two clusters in this cluster analysis had the highest silhouette width of 0.327. NA = normal axis; RAD = Right axis deviation; LAD = Left Axis Deviation; EAD: extreme axis deviation; LBBB: Left bundle branch block pattern; RBBB = Right bundle branch block pattern.

**Table 2:** Cluster analysis on all PVCs with the RBBB pattern based on only numerical variables using partitioning around mediod (left) and Simple K-means (right).

	Partitioning around Mediod			Simple K-Means	
	Cluster 1	Cluster 2	*p*	Cluster 1	Cluster 2
V1S	0.50 (0.00–1.50)	0.55 (0.00–1.13)	0.0374	0.50	1.67
V2S	1.38 (1.00–2.69)	1.00 (0.50–1.65)	0.133	1.35	3.07
V1R	5.38 (3.13–7.19)	3.25 (2.25–4.63)	0.0003287	3.83	7.56
V2R	9.00 (7.00–11.94)	4.00 (3.00–5.00)	2.94 × 10^−14^	5.91	11.04
V2R Sinus	3.38 (1.63–4.25)	1.00 (0.68–2.50)	3.24 × 10^−06^	1.72	4.94
V2 Total PVC	10.12 (8.85–13.50)	5.50 (4.00–6.85)	2.82 × 10^−11^	7.14	14.12
V2 Total Sinus	9.75 (7.60–12.50)	7.25 (4.88–9.33)	8.987 × 10^−05^	7.79	13.24
V3R	7.50 (6.00–9.94)	2.90 (2.00–4.00)	2.13 × 10^−11^	5.30	8.94
Transition Lead	No transition lead: 1 V2.5: 2 V3.5: 9 V4.0: 1 V4.5: 11 V5.0: 12 V5.5: 9 V6.0: 5	V1.5: 5 V2.0: 2 V2.5: 10 V3.0: 1 V3.5: 12 V4.0: 2 V4.5: 1 V5.0: 4 V5.5: 4 V6.0: 2			
Frontal Plane Axis	NA: 0 (0) RAD: 11 (22) LAD: 25 (50) EAD: 12 (24) +90: 0 (0) −30: 0 (0) −90: 2 (4) +180: 0 (0)	NA: 1 (2.3) RAD: 11 (25.6) LAD: 16 (37.2) EAD:12 (27.9) +90: 0 (0) −30: 0 (0) −90: 3 (7.0) +180: 0 (0)			
Average percentage of RVOT based on 3 equations	0	7.0	0.0005		

Values were reported as median (Q1–Q3) or count (%). The number of clusters is based on the relative silhouette width. Two clusters in this cluster analysis had the highest silhouette width of 0.261. NA = normal axis; RAD = right axis deviation; LAD = left axis deviation; EAD = extreme axis deviation.

**Table 3:** Cluster analysis on all PVCs with the LBBB pattern using only numerical variables using Partitioning around Mediod (left) and Simple K-means (right).

	Partitioning around Mediod			Simple K-Means	
	Cluster 1	Cluster 2	*p*	Cluster 1	Cluster 2
V1S	3.75 (2.19–5.31)	7.25 (6.00–9.00)	<2.2 × 10^−16^	3.53	7.60
V2S	3.75 (1.69–5.56)	10.50 (8.50–13.00)	<2.2 × 10^−16^	3.65	11.23
V1R	0.50 (0.00–1.56)	0.75 (0.10–1.25)	0.252	1.23	0.81
V2R	2.75 (1.00–4.50)	1.5 (0.50–2.75)	0.00037	3.44	1.60
V2R Sinus	1.50 (1.00–3.00)	1.75 (1.00–3.00)	0.4057	2.08	2.18
V2 Total PVC	6.375 (5.00–8.50)	12.50 (10.50–15.00)	<2.2 × 10^−16^	7.09	12.84
V2 Total Sinus	7.00 (5.25–9.00)	9.00 (5.90–10.50)	0.001081	7.66	8.53
V3R	5.50 (3.00–7.13)	2.25 (1.13–3.50)	7.54 × 10^−10^	5.94	2.31
Transition Lead	No transition lead: 1 V1.0: 4 V1.5: 26 V2.0: 10 V2.5: 23 V3.0: 3 V3.5: 6 V4.0: 4 V4.5: 5 V5.5: 1 V6.0: 1	V2.0: 2 V2.5: 10 V3.0: 4 V3.5: 32 V4.0: 8 V4.5: 10 V5.0: 5 V5.5: 6 V6.0: 2			
Frontal Plane Axis	NA: 57 (67.9) RAD: 9 (10.7) LAD:14 (16.7) EAD: 0 (0) +90: 3 (3.6) −30: 1 (1.2) −90: 0 (0) +180: 0 (0)	NA: 49 (62.0) RAD: 15 (19.0) LAD: 10 (16.7) EAD: 0(0) +90: 5 (6.3) −30: 0 (0) −90: 0 (0) +180: 0 (0)			
Average percentage of RVOT based on 3 equations	36.4	82.7	5.39e^−25^		

Values were reported as median (Q1–Q3) or count (%). The number of clusters is based on the relative silhouette width. Two clusters in this cluster analysis had the highest silhouette width of 0.314. NA = normal axis; RAD = right axis deviation; LAD = left axis deviation; EAD = extreme axis deviation.

**Table 4:** Cluster analysis on all PVCs with the LBBB pattern and inferior axis using partitioning around mediods based on only numerical variables.

	Cluster 1	Cluster 2	*p*
V1S	7.25 (6.00–9.25)	4.25 (1.50–5.950)	8.04 × 10^−13^
V2S	10.5 (8.50–13.00)	5.00 (2.50–6.30)	<2.2 × 10^−16^
V1R	0.80 (0.25–1.50)	1.00 (0.00–2.00)	0.224
V2R	1.75 (0.90–2.90)	2.5 (1.10–4.00)	0.035
V2R Sinus	1.75 (1.00–3.00)	1.5 (1.00–3.00)	0.7468
V2 Total PVC	12.75 (11.00–15.00)	7.50 (5.63–9.13)	2.356 × 10^−15^
V2 Total Sinus	9.00 (6.00–11.00)	6.75 (4.75–8.375)	0.002283
V3R	2.50 (1.50–4.00)	6.00 (3.5–7.88)	<2.384 × 10^−14^
Transition Lead	V2.0: 2 V2.5: 7 V3.0: 2 V3.5: 28 V4.0: 5 V4.5: 5 V5.0: 2 V5.5: 2	No transition lead: 1 V1.0: 3 V1.5: 9 V2.0: 8 V2.5: 19 V3.0: 4 V3.5: 4 V4.0: 2 V6.0: 1	
Frontal Plane Axis	NA: 35 () RAD: 15 () LAD: 0 (0) EAD: 0 (0) +90: 3 () −30: 0 (0) −90: 0 (0) +180: 0 (0)	NA: 37 (72.5) RAD: 9 (17.6) LAD: 0 (0) EAD: 0 (0) +90: 5 (9.8) −30: 0 (0) −90: 0 (0) +180: 0 (0)	
Average percentage of RVOT based on 3 equations	81.4	40.5	1.65e^−13^

Values were reported as median (Q1–Q3) or count (%). The number of clusters is based on the relative silhouette width. Two clusters in this cluster analysis had the highest silhouette width of 0.281. NA = normal axis; RAD = right axis deviation; LAD = left axis deviation; EAD = extreme axis deviation.

**Table 5:** Cluster analysis on all PVCs with the LBBB pattern and superior axis using partitioning around mediods based on only numerical variables.

	Cluster 1	Cluster 2	*p*
V1S	3.75 (2.30–5.10)	8.00 (6.81–10.88)	2.45 × 10^−8^
V2S	2.50 (1.00–5.00)	13.03 (0.03–1.00)	1.82 × 10^−15^
V1R	0.10 (0.00–0.75)	0.50 (0.03–1.00)	0.9878
V2R	2.00 (0.50–4.50)	0.88 (0.25–2.36)	0.0242
V2R Sinus	1.50 (1.00–2.75)	1.75 (1.00–3.44)	0.07786
V2 Total PVC	6.00 (4.50–7.90)	13.55 (11.56–15.88)	6.91 × 10^−12^
V2 Total Sinus	7.50 (6.00–9.00)	9.50 (8.25–10.50)	0.002145
V3R	4.50 (1.75–6.25)	1.25 (0.56–2.62)	9.74 × 10^−10^
Transition Lead	V1.0: 1 V1.5: 17 V2.0: 2 V2.5: 6 V3.0: 1 V3.5: 4 V4.0: 2 V4.5: 5 V5.5: 2 V6.0: 1	V2.5: 1 V3.5: 2 V4.0: 3 V4.5: 5 V5.0: 3 V5.5: 3 V6.0: 1	
Frontal Plane Axis	NA: 23 (56.1) RAD: 0 (0) LAD: 17 (41.5) EAD: 0 (0) +90:0 (0) −30: 1 (2.4) −90: 0 (0) +180: 0 (0)	NA: 11 (61.1) RAD: 0 (0) LAD: 7 (38.9) EAD: 0 (0) +90:0 (0) −30: 0 (0) −90: 0 (0) +180: 0 (0)	
Average percentage of RVOT based on all 3 equations	40.5	86.3	2.28e^−13^

Values were reported as median (Q1–Q3) or count (%). The number of clusters is based on the relative silhouette width. Two clusters in this cluster analysis had the highest silhouette width of 0.399. NA = normal axis; RAD = right axis deviation; LAD = left axis deviation; EAD = extreme axis deviation.

## Data Availability

The data cannot be shared.

## Supplementary Materials

The following supporting information can be downloaded at: https://www.mdpi.com/article/10.3390/jcm12175558/s1, Figure S1. This shows the results of cluster analysis using only numerical variables and no categorical variables. (Bajaj. Bennett, and Rabkin 2023); Figure S2. This shows the results of cluster analysis using only numerical variables in PVC with a RBBB configuration (Bajaj. Bennett, and Rabkin 2023); Figure S3. This shows the results of cluster analysis using only numerical variables in PVC with a LBBB configuration (Bajaj. Bennett, and Rabkin 2023); Table S1: ECG characteristics of PVCs from 338 ECGs; Table S2: Cluster analysis on the entire sample using Partitioning around Medoids based on both numerical and categorical variables; Table S3: Cluster analysis on all PVCs with LBBB pattern and inferior axis using Partitioning around Medoids based on only numerical variables; Table S4: Cluster analysis on all PVCs with LBBB pattern and superior axis using Partitioning around Medoids based on only numerical variables; Table S5a: Cluster analysis on all PVCs based on simple K-means clustering with only numerical variables; Table S5b: Cluster analysis on all PVCs with LBBB pattern based on simple K means clustering with only numerical variables.; Table S5c: Cluster analysis on all PVCs with RBBB pattern based on simple K means clustering with only numerical variables.

## Abbreviations

PVC: premature ventricular complexes; BBB: Bundle branch block; LVOT: left ventricular outflow tract; TL: transitional (precordial) lead; RVOT: right ventricular outflow tract; FPA: frontal plan axis.
