# Supplementary material for: Identifying Premature Ventricular Complexes from Outflow Tracts Based on PVC Configuration: A Machine Learning Approach
Source: J Clin Med. 2023 Aug 26;12(17):5558. doi: 10.3390/jcm12175558 (PMC10487978; doi:10.3390/jcm12175558)
Supplement: Supplementary file 1 [file jcm-12-05558-s001.zip › jcm-2528322-supplementary.pdf]

SUPPLEMENT

Identifying premature ventricular complexes from outflow tracts based on PVC configuration: A machine learning approach

Short title: PVC configuration

Sargun Bajaj

Matthew T. Bennett

Simon W. Rabkin

ABSTRACT

Problem: Current inferences about the site of origin (SOO) of premature ventricular complexes (PVC) from the surface ECG have not been subject to newer data analytic techniques that identify signals that are not recognized by visual inspection. ***Aim***: to apply data analytics to PVC characteristics. ***Methods***: PVCs from 12 lead ECGs of a consecutive series of 338 individuals were examined by unsupervised machine learning cluster analysis and indexes were compared to a composite criteria for SOO. ***Results:*** V1S plus V2S ≤ 9.25 of the PVC had a LVOT origin (sensitivity 95.4%; specificity 97.5%). V1R+V2R+V3R>15.0 (a RBBB configuration) likely had a LVOT origin. For PVCs with V1S plus V2S > 12.75 (LBBB configuration) likely had a RVOT origin. PVC with V1S plus V2S > 14.25 (LBBB configuration) and all inferior leads positive, likely had a RVOT origin. ***Conclusions***: Newer data analytic techniques provide a non-invasive approach to identify PVC SOO.

Key words: Premature ventricular complexes; PVC morphology, outflow tract origin; cluster analysis, unsupervised machine learning

Abbreviations:

PVC: Premature ventricular complexes

BBB: Bundle branch block

LVOT: left ventricular outflow tract

TL: Transitional (precordial) lead

RVOT: right ventricular outflow tract

FPA: Frontal plan axis

Plain language summary:

We used an artificial intelligence strategy called unsupervised machine learning cluster analysis and applied it to extra beats coming from the lower chambers of the heart – the ventricles. This data analysis strategy identified two different types of extra beats. Measuring the height (positive deflection) and the depth (negative deflection) allowed us to identify where the extra beat originated. Accurate localization of the extra beat, will allow clinicians to counsel patients regarding the risk and success of attempts to eliminate the site of origin of the extra beats.

Tweetable abstract

Unsupervised machine learning cluster analysis performed on PVC characteristics yielded two main clusters and characteristics that identified site of origin e.g. V1S plus V2S ≤ 9.25 had a very high probability of a LVOT origin

SUMMARY POINTS

- The presence of a high PVC burden is a recommendation for PVC ablation if PVCs cannot be managed by antiarrhythmic drug therapy, especially if there is a single dominant PVC morphology
- Accurate PVC location ascertainment will allow clinicians to counsel patients regarding the risk and success of ablation
- PVC localization to the RV outflow tract is easier for ablation while some LVOT outflow tract ablations have a greater risk for damage to the aortic valve or left main coronary artery.
- Unsupervised machine learning cluster analysis was performed on PVC characteristics from 338 different patient ECGs and it yielded two main clusters
- Comparing machine learning clusters to a composite of three different previously reported PVC characteristics for site of origin of PVCs found:
- V1S plus V2S ≤ 9.25 of the PVC had a very high probability of a LVOT origin
- V1R+V2R+V3R>15.0 of the PVC likely had a LVOT origin.
- V1S plus V2S > 12.75 of the PVC likely had a RVOT origin
- V1S plus V2S > 14.25 and all inferior leads positive of the PVC, likely had a RVOT origin.

The characteristics of the PVCs from each of the 12 lead ECGs were assessed and outlined in Table 1.

Table S1. ECG characteristics of PVCs from 338 ECGs

PVC Characteristics	N= 338[1]	N= 338[2]
Frontal Plan Axis		
Normal axis (NA)	127 (37.6)	127 (37.6)
Left Axis deviation (LAD)	93 (27.5)	93 (27.5)
Right Axis deviation (RAD)	70 (20.7)	70 (20.7)
Extreme axis deviation (EAD)	24 (7.1)	24 (7.1)
90 degrees (Borderline RAD)	15 (4.4)	15 (4.4)
-90 degrees (Borderline EAD)	5 (1.5)	5 (1.5)
-30 degrees (Borderline LAD)	3 (0.9)	3 (0.9)
180 degrees (Borderline EAD)	1 (0.3)	1 (0.3)
Transition Lead		
V1.0	5 (1.5)	5 (1.5)
V1.5	31 (9.2)	31 (9.2)
V2.0	14 (4.1)	14 (4.1)
V2.5	45 (13.3)	45 (13.3)
V3.0	8 (2.4)	8 (2.4)
V3.5	59 (17.5)	59 (17.5)
V4.0	15 (4.4)	15 (4.4)
V4.5	27 (8.0)	27 (8.0)
V5.0	21 (6.2)	21 (6.2)
V5.5	20 (5.9)	20 (5.9)
V6.0	10 (3.0)	10 (3.0)
No transition lead	83 (24.6)	83 (24.6)
Bundle Branch Block Pattern		
Left Bundle Branch Block	163 (48.2)	163 (48.2)
Right Bundle Branch Block	93 (27.5)	93 (27.5)
Posterior ‘pattern	64 (18.9)	64 (18.9)
Anterior ‘pattern	18 (5.3)	18 (5.3)
V1S PVC complex amplitude	3.25 (3.33)	2.00 (0.26 – 5.86)
V2S PVC complex amplitude	4.74 (4.76)	2.75 (1.00 - 7.44)
V1R PVC complex amplitude	2.57 (2.71)	1.90 (0.50 - 3.79)
V2R PVC complex amplitude	2.23 (3.91)	1.78 (1.00 – 3.00)
V3R PVC complex amplitude	5.41 (4.13)	4.50 (2.25 – 7.94)
V2 PVC complex amplitude	9.30 (4.56)	8.78 (6.00 – 11.75)
V2R/(V2R + V2S) PVC complex	0.53 (0.36)	0.62 (0.16 – 0.87)

[1]Values were reported as mean (SD) for numerical variables or count (%) for categorical variables. [2] Values were reported as median (Q1-Q3) or count (%).

Cluster analysis on the entire sample using Partitioning around Medoids based on both numerical and categorical variables.

We performed a cluster analysis utilizing Partitioning around Medoids for the entire sample using on both numerical and categorical variables**.** Three clusters were evident but two of them (cluster 1 and 3 were not significantly different from each other (Table S2).

Table S2. Cluster analysis on the entire sample using Partitioning around Medoids based on both numerical and categorical variables.

	Cluster 1	Cluster 2	Cluster 3	P (overall)
V1S	0.50 (0.00 – 1.50)	6.00 (4.25 – 7.69)	0.50 (0.00 – 1.50)	<2e-16*
V2S	1.50 (1.00 – 4.00)	7.50 (4.75 – 11.00)	1.00 (0.50 – 2.00)	<2e-16*
V1R	3.00 (1.81 – 4.75)	0.5 (0.00 – 1.50)	3.78 (2.06 – 6.00)	<2e-16*
V2R	5.63 (2.25- 8.08)	1.75 (0.75 – 3.44)	6.13 (4.25 – 9.25)	<2e-16*
V2R Sinus	2.00 (1.00 – 3.00)	1.50 (1.00 – 2.98)	2.25 (1.00 – 3.50)	0.408
V2 Total PVC	8.50 (5.81 – 10.00)	9.75 (6.81 – 12.94)	7.78 (5.50 – 10.25)	0.00244*
V2 Total Sinus	7.38 (5.75 – 9.50)	7.63 (5.50 – 10.00)	8.50 (6.31 – 10.79)	0.068
V3R	7.25 (3.81 – 10.24)	3.00 (1.75 – 5.75)	5.10 (3.00 – 8.38)	<7.3e-10*
Transition Lead	No transition lead: 82<br>V1.0: 2<br>V1.5: 2<br>V2.0: 5<br>V2.5: 2<br>V4.0: 1<br>V5.0: 2<br>V5.5: 1<br>V6.0: 1<br>Other: 5	No transition lead: 1<br>V1.0: 2<br>V1.5: 21<br>V2.0: 9<br>V2.5: 31<br>V3.0: 6<br>V3.5: 39<br>V4.0: 11<br>V4.5: 13<br>V5.0: 5<br>V5.5: 6<br>V6.0: 2	V1.0: 1<br>V1.5: 8<br>V2.5: 12<br>V3.0: 2<br>V3.5: 20<br>V4.0:3<br>V4.5: 14<br>V5.0: 14<br>V5.5: 13<br>V6.0: 7	
Frontal Plane Axis	NA: 20 (20.4)<br>RAD: 41 (41.8)<br>LAD: 27 (27.6)<br>EAD: 0 (0)<br>+90: 7 (7.1)<br>-30: 2 (2.0)<br><br>-90: 0 (0)<br>+180:1 (1.0)	NA: 106 (72.6)<br>RAD: 17(11.6)<br>LAD: 14 (9.6)<br>EAD: 0 (0)<br>+90: 8 (5.5)<br>-30:1 (0.7)<br>-90: 0 (0)<br>+180: 0 (0)	NA: 1 (1.1)<br>RAD: 12 (12.8)<br>LAD: 52 (55.3)<br>EAD: 24 (25.5)<br>+90: 0 (0)<br>-30: 0 (0)<br>-90: 5 (5.3)<br>+180: 0 (0)	
BBB	LBBB: 8 (8.2)<br>RBBB: 9 (9.2)<br>Posterior: 64 (65.3)<br>Anterior: 17 (17.3)	LBBB: 144 (98.0)<br>RBBB=1 (0.7)<br>Posterior=1 (0.7)<br>Anterior: 1 (0.7)	LBBB: 11 (11.7)<br>RBBB: 83 (88.3)<br><br>Posterior: 0 (0)	

			Anterior: 0 (0)	
RVOT based on Kaypakli *et al.*	21 (21.4%)	125 (85.6)	5 (5.4)	<2e-16**
RVOT based on Betenski *et al*.	11 (11.5%)	68 (46.6)	1 (1.1)	<2e-16**
RVOT based on Yoshida *et al.*	14 (14.9%)	77 (52.7)	4 (4.3)	<2e-16**
Average percentage of RVOT based on 3 equations	16.0%	61.6	3.6	<2e-16**

Values were reported as median (Q1-Q3) or count (%). Number of clusters are based on the relative silhouette width. 3 clusters in this cluster analysis had a highest silhouette width of 0.311. *Significant difference Between Cluster 1 and 2 & Cluster 2 and 3.
** Significant difference between all 3 groups. I.e. each cluster is significantly different from other 2 clusters. Significance based on ANOVA.NA= normal axis; RAD= Right axis deviation; LAD= Left Axis Deviation; EAD: extreme axis deviation; LBBB: Left bundle branch block pattern; RBBB= Right bundle branch block pattern. According to Kaypakli et al., (V1S – V2S) - (V1R + V2R) > 1.625 predicts RVOT. According to Yoshida et al., V2 transition ratio ≥ 0.6 predicts LVOT. According to Betenski et al., V2S/V3R index ≤ 1.5.

Cluster analysis of LBBB pattern PVCs with inferior axis or superior axis

Cluster analysis of PVC sample with LBBB pattern and all inferior leads positive without any categorical variables was performed (Table S3). This cluster analysis produced 2 clusters as this number of clusters had the highest silhouette width of 0.281. Six out of 8 numerical variables were significantly (p<0.05) different between the 2 clusters. Cluster 1 had majority RVOT (81.4%) and Cluster 2 had majority LVOT origin (59.5%). To determine cluster assignment, sum of V1S and V2S > 14.25 can be used to predict assignment to cluster 1 or a high probability of RVOT origin with a sensitivity of 90.6% and a specificity of 100%.

Cluster analysis of PVC sample with LBBB pattern and not all inferior leads positive without any categorical variables produced 2 clusters as this number of clusters had the highest silhouette width of 0.399 (Table S4). Six out of 8 numerical variables were significantly different between the 2 clusters (p<0.05). Cluster 1 had majority LVOT (59.5%) and Cluster 2 had majority RVOT origin (86.3%). To determine cluster assignment, PVC with a high probability of RVOT origin (Cluster 2) was characterized by the formula V1S + V2S – V3R ≤ 11.5 had a 94.4 % sensitivity and 97.6 % specificity.

Table S3. Cluster analysis on all PVCs with LBBB pattern and inferior axis using Partitioning around Medoids based on only numerical variables.

	Cluster 1	Cluster 2	P
V1S	7.25 (6.00 – 9.25)	4.25 (1.50 – 5.950)	8.04e-13
V2S	10.5 (8.50 – 13.00)	5.00 (2.50 – 6.30)	<2.2e-16
V1R	0.80 (0.25 – 1.50)	1.00 (0.00 – 2.00)	0.224
V2R	1.75 (0.90 – 2.90)	2.5 (1.10 – 4.00)	0.035
V2R Sinus	1.75 (1.00 – 3.00)	1.5 (1.00 – 3.00)	0.7468
V2 Total PVC	12.75 (11.00 – 15.00)	7.50 (5.63 – 9.13)	2.356e-15
V2 Total Sinus	9.00 (6.00 – 11.00)	6.75 (4.75 - 8.375)	0.002283
V3R	2.50 (1.50 – 4.00)	6.00 (3.5 – 7.88)	<2.384e-14
Transition Lead	V2.0: 2<br>V2.5: 7<br>V3.0: 2<br>V3.5: 28<br>V4.0: 5<br>V4.5: 5<br>V5.0: 2<br>V5.5: 2	No transition lead: 1<br>V1.0: 3<br>V1.5: 9<br>V2.0: 8<br>V2.5: 19<br>V3.0: 4<br>V3.5: 4<br>V4.0: 2<br>V6.0: 1	
Frontal Plane Axis	NA: 35 ()<br>RAD: 15 ()<br>LAD: 0 (0)<br>EAD: 0 (0)<br>+90: 3 ()<br>-30: 0 (0)<br>-90: 0 (0)<br>+180: 0 (0)	NA: 37 (72.5)<br>RAD: 9 (17.6)<br>LAD: 0 (0)<br>EAD: 0 (0)<br>+90: 5 (9.8)<br>-30: 0 (0)<br>-90: 0 (0)<br>+180: 0 (0)	
Average percentage of RVOT based on 3 equations	81.4	40.5	1.65e-13

Values were reported as median (Q1-Q3) or count (%). Number of clusters are based on the relative silhouette width. 2 clusters in this cluster analysis had a highest silhouette width of 0.281. NA= normal axis; RAD= Right axis deviation; LAD= Left Axis Deviation; EAD: extreme axis deviation. P value is based on simple t-tests.

Table S4. Cluster analysis on all PVCs with LBBB pattern and superior axis using Partitioning around Medoids based on only numerical variables.

	Cluster 1	Cluster 2	P
V1S	3.75 (2.30 – 5.10)	8.00 (6.81 – 10.88)	2.45e-08
V2S	2.50 (1.00 – 5.00)	13.03 (0.03 – 1.00)	1.82e-15
V1R	0.10 (0.00 – 0.75)	0.50 (0.03 – 1.00)	0.9878
V2R	2.00 (0.50 – 4.50)	0.88 (0.25 – 2.36)	0.0242
V2R Sinus	1.50 (1.00 – 2.75)	1.75 (1.00 – 3.44)	0.07786
V2 Total PVC	6.00 (4.50 – 7.90)	13.55 (11.56 – 15.88)	6.91e-12
V2 Total Sinus	7.50 (6.00 – 9.00)	9.50 (8.25 – 10.50)	0.002145
V3R	4.50 (1.75 – 6.25)	1.25 (0.56 – 2.62)	9.74e-10
Transition Lead	V1.0: 1<br>V1.5: 17<br>V2.0: 2<br>V2.5: 6<br>V3.0: 1<br>V3.5: 4<br>V4.0: 2<br>V4.5: 5<br>V5.5: 2<br>V6.0: 1	V2.5: 1<br>V3.5: 2<br>V4.0: 3<br>V4.5: 5<br>V5.0: 3<br>V5.5: 3<br>V6.0: 1	
Frontal Plane Axis	NA: 23 (56.1)<br>RAD: 0 (0)<br>LAD: 17 (41.5)<br>EAD: 0 (0)<br>+90:0 (0)<br>-30: 1 (2.4)<br>-90: 0 (0)<br>+180: 0 (0)	NA: 11 (61.1)<br>RAD: 0 (0)<br>LAD: 7 (38.9)<br>EAD: 0 (0)<br>+90:0 (0)<br>-30: 0 (0)<br>-90: 0 (0)<br>+180: 0 (0)	
Average percentage of RVOT based on all 3 equations	40.5	86.3	2.28e-13

Values were reported as median (Q1-Q3) or count (%). Number of clusters are based on the relative silhouette width. 2 clusters in this cluster analysis had a highest silhouette width of 0.399. NA= normal axis; RAD= Right axis deviation; LAD= Left Axis Deviation; EAD: extreme axis deviation. P value is based on simple t-tests.

Cross validation of Partition around Methods Cluster Analysis

In order to cross validate the Cluster analysis that used the Partition around Mediods method, we used another machine learning methodology namely Weka that partitions the observations into k clusters with each observation being in a cluster based on its proximity to the cluster center (K means) [16].

The number of PVCs in each cluster using both PAM clustering and Simple K-means were not significantly different (p=0.23). Additionally, average values for each of the 8 numerical variables in both clusters were not significantly (p=0.46) different between simple K means clustering and PAM clustering. Clusters found using simple K means clustering were again similar to clusters found using Partitioning around medoids (Table S5b). The number of PVCs in each cluster using both PAM clustering and Simple K-means was not significantly different (p=0.91). Additionally, average values for each of the 8 numerical variables in both clusters were not significantly (p=0.84) different between simple K means clustering and PAM clustering.

Table S5a. Cluster analysis on all PVCs based on simple K means clustering with only numerical variables.

	Cluster 1*	Cluster 2**
V1S	6.71	0.98
V2S	9.33	1.73
V1R	0.73	3.78
V2R	1.70	6.48
V2R Sinus	2.14	2.29
V2 Total PVC	11.03	8.16
V2 Total Sinus	8.54	8.23
V3R	2.77	7.15

Table S5b. Cluster analysis on all PVCs with LBBB pattern based on simple K means clustering with only numerical variables.

	Cluster 1$^{\#}$	Cluster 2$^{\#\#}$
V1S	3.53	7.60
V2S	3.65	11.23
V1R	1.23	0.81
V2R	3.44	1.60
V2R Sinus	2.08	2.18
V2 Total PVC	7.09	12.84
V2 Total Sinus	7.66	8.53
V3R	5.94	2.31

Table S5c. Cluster analysis on all PVCs with RBBB pattern based on simple K means clustering with only numerical variables.

	Cluster 1[£]	Cluster 2[££]
V1S	0.50	1.67
V2S	1.35	3.07
V1R	3.83	7.56
V2R	5.91	11.04
V2R Sinus	1.72	4.94
V2 Total PVC	7.14	14.12
V2 Total Sinus	7.79	13.24
V3R	5.30	8.94

[1]Values expressed as mean. *n=204; **n=134; [#]n=83; [##]n=80; [£] n=72; [££]n=21.

Supplementary Figures

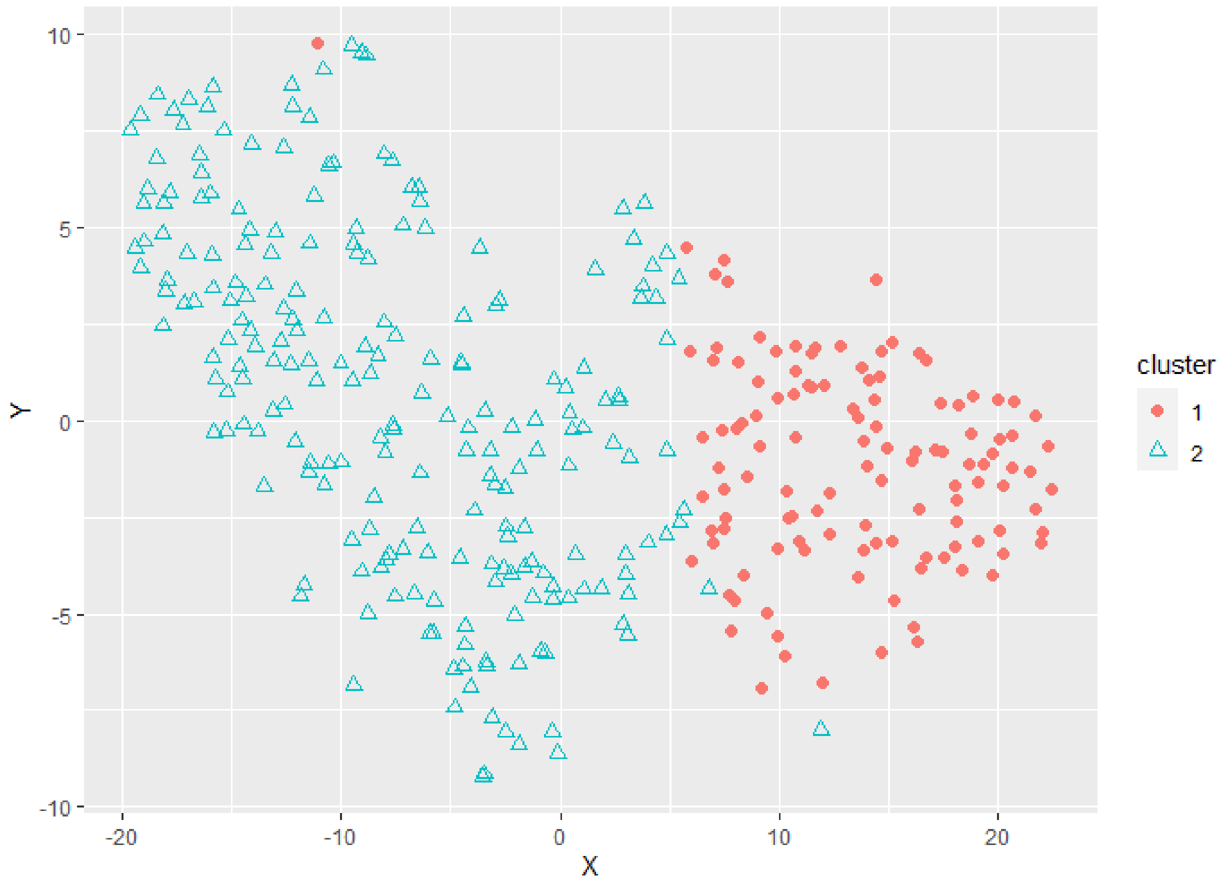

Figure S1. This shows the results of cluster analysis using only numerical variables and no categorical variables. (Bajaj. Bennett, and Rabkin 2023)

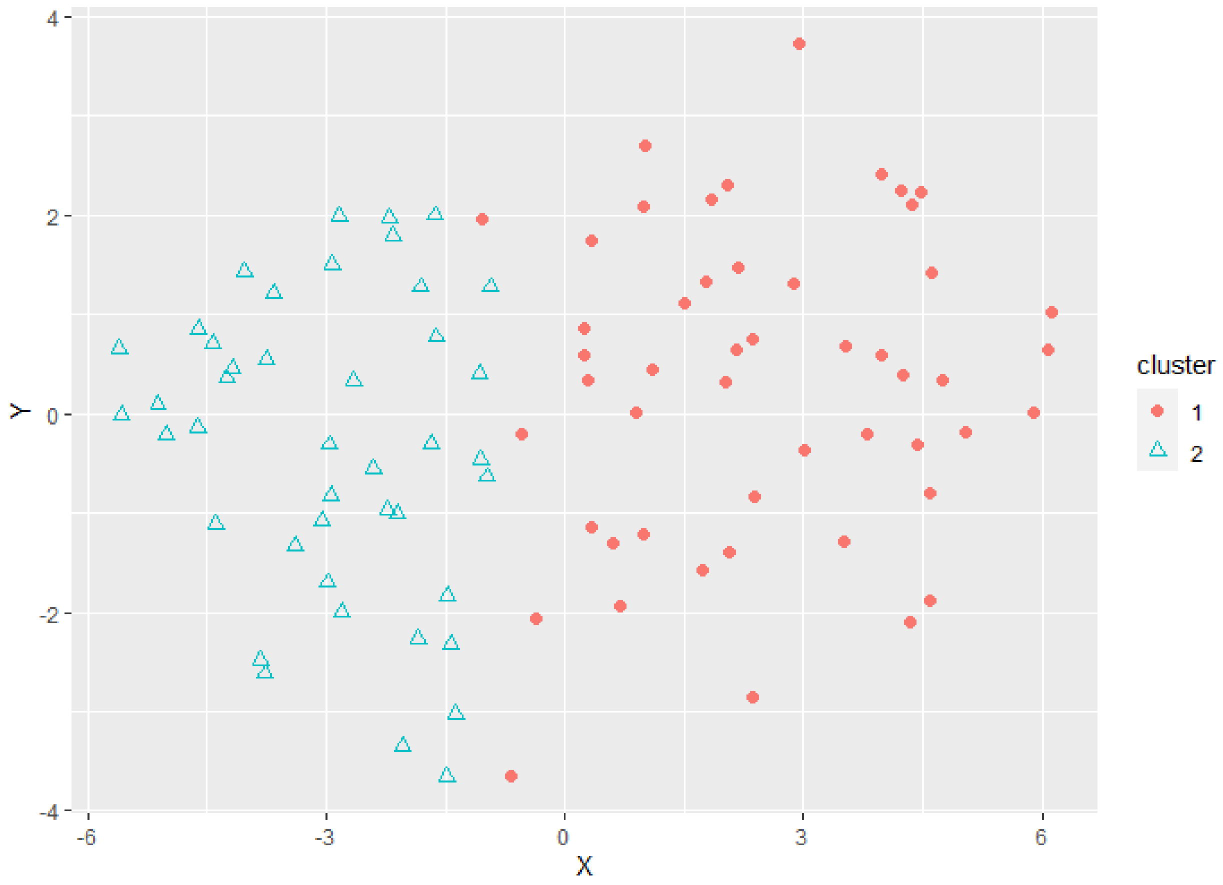

Figure S2. This shows the results of cluster analysis using only numerical variables in PVC with a RBBB configuration (Bajaj. Bennett, and Rabkin 2023)

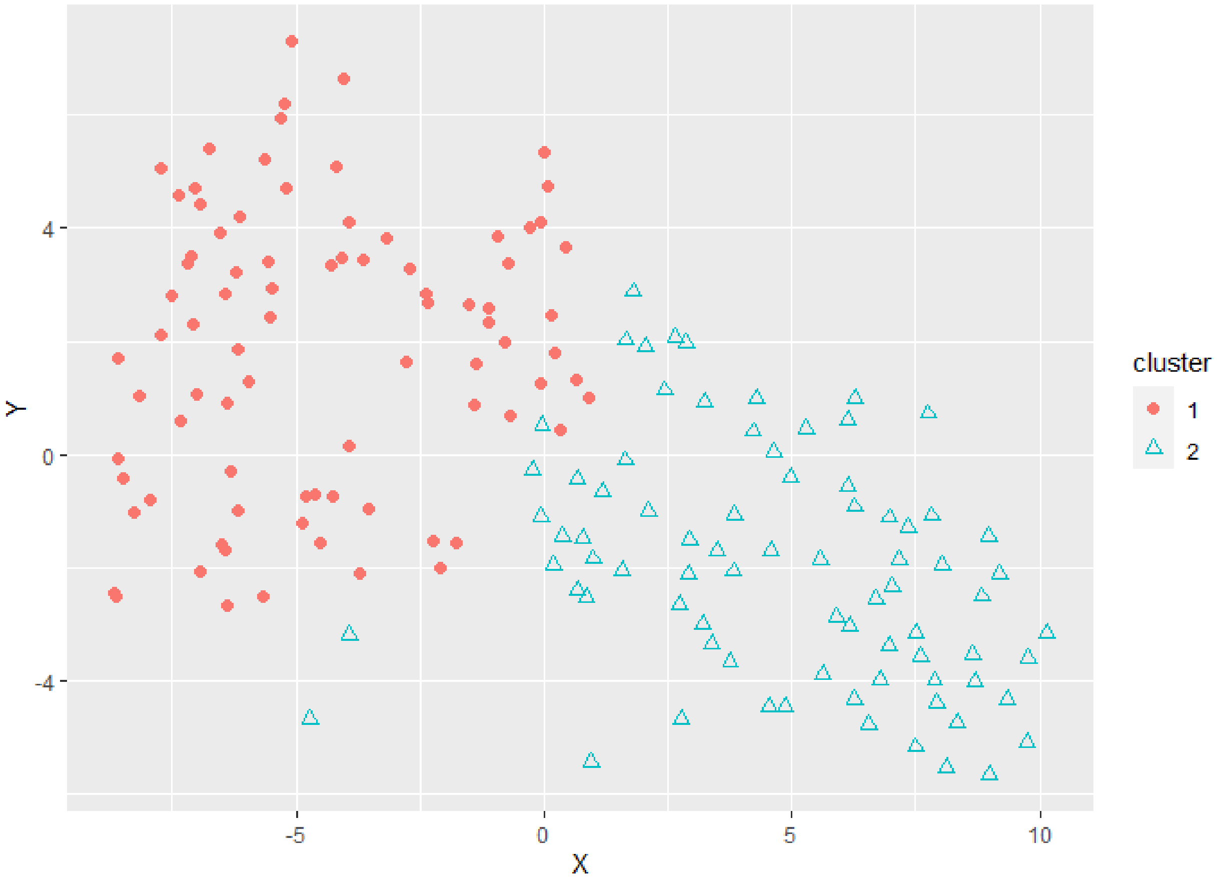

Figure S3. This shows the results of cluster analysis using only numerical variables in PVC with a LBBB configuration (Bajaj. Bennett, and Rabkin 2023)
